# Integrative genome, transcriptome, microRNA, and degradome analysis of water dropwort (*Oenanthe javanica*) in response to water stress

**DOI:** 10.1038/s41438-021-00707-8

**Published:** 2021-12-01

**Authors:** Jie-Xia Liu, Qian Jiang, Jian-Ping Tao, Kai Feng, Tong Li, Ao-Qi Duan, Hao Wang, Zhi-Sheng Xu, Hui Liu, Ai-Sheng Xiong

**Affiliations:** State Key Laboratory of Crop Genetics and Germplasm Enhancement, Ministry of Agriculture and Rural Affairs Key Laboratory of Biology and Germplasm Enhancement of Horticultural Crops in East China, College of Horticulture, Nanjing Agricultural University, 1 Weigang, 210095 Nanjing, China

## Abstract

Water dropwort (Liyang Baiqin, *Oenanthe javanica* (BI.) DC.) is an aquatic perennial plant from the Apiaceae family with abundant protein, dietary fiber, vitamins, and minerals. It usually grows in wet soils and can even grow in water. Here, whole-genome sequencing of *O. javanica* via HiSeq 2000 sequencing technology was reported for the first time. The genome size was 1.28 Gb, including 42,270 genes, of which 93.92% could be functionally annotated. An online database of the whole-genome sequences of water dropwort, Water dropwortDB, was established to share the results and facilitate further research on *O. javanica* (database homepage: http://apiaceae.njau.edu.cn/waterdropwortdb). Water dropwortDB offers whole-genome and transcriptome sequences and a Basic Local Alignment Search Tool. Comparative analysis with other species showed that the evolutionary relationship between *O. javanica* and *Daucus carota* was the closest. Twenty-five gene families of *O. javanica* were found to be expanded, and some genetic factors (such as genes and miRNAs) related to phenotypic and anatomic differentiation in *O. javanica* under different water conditions were further investigated. Two miRNA and target gene pairs (miR408 and *Oja15472*, miR171 and *Oja47040*) were remarkably regulated by water stress. The obtained reference genome of *O. javanica* provides important information for future work, thus making in-depth genetic breeding and gene editing possible. The present study also provides a foundation for the understanding of the *O. javanica* response to water stress, including morphological, anatomical, and genetic differentiation.

## Introduction

**Fig. 1:**
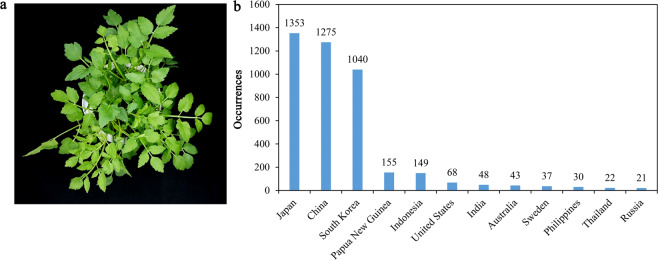
Georeferenced records and an image of *O. javanica*. **a** Image of *O. javanica* cv. “Liyang Baiqin”. **b** Georeferenced occurrence records of *O. javanica* (BI.) DC. on the world (the country with occurrence records ≥20 is shown). The data were obtained in 2021 from the GBIF website (www.gbif.org)

Water dropwort (Liyang Baiqin, *Oenanthe javanica* (BI.) DC.) is an aquatic perennial herb that belongs to the Apiaceae family. Different chromosome numbers for *O. javanica* have been reported by different researchers, such as 22, 42, and 44^1–3^. Apiaceae vegetables with high nutritional and economic value, such as carrot, celery and parsley, are commonly used and consumed^4–6^. *O. javanica* is widely cultivated in East Asian countries, such as China, Japan, and South Korea (Fig. 1). The evolutionary branch of the genus *Oenanthe* was divided from the tribe Oenantheae ~11 million years ago, and then *O. javanica* emerged approximately 4.73 million years ago (http://www.timetree.org).

*O. javanica* is a vegetable with high vitamin and mineral contents. It has several pharmacological properties, including hepatoprotective^7^, antithrombotic, antiarrhythmic, antidiabetic, antihepatitis B virus^8^, neuroprotective^9^, and anticancer activities^10^. In traditional Chinese medicine, *O. javanica* is used to treat jaundice, hypertension, fever, abdominal pain, leucorrhea, mumps, and urinary difficulties^11^. Considerable pharmacological studies have been performed on *O. javanica*, but its genetic information is still limited. Our laboratory group has created transcriptome and small RNA databases of *O. javanica* and conducted some studies related to abiotic stress on the basis of these databases^12–14^.

Water plays a critical role in plant growth and development. Given the detrimental effects that waterlogging or water deficit has on plants, water conditions have been one of the most important constraints affecting crops, vegetables, forages, and other plant production worldwide^15,16^. The amount of molecular oxygen in water is much lower than that in air. For aerobic organisms, soil waterlogging may cause the plants to become hypoxic or anoxic and has been a challenge for plant growth and development^17^. Water stress affects nearly every aspect of plant physiology and metabolism, and numerous changes that occur under water stress have been reported^15,18^. As an aquatic vegetable, the physiological performance of *O. javanica* in humid and waterlogged conditions may be different from that of xerophytes.

The regulatory factors that respond to different water conditions are unknown in *O. javanica*. miRNAs are well known to be involved in a series of physiological processes, including growth, development, and biotic and abiotic stress responses^19^. They bind to mRNAs at specific sequences and inhibit the expression of targets by targeting the mRNA for degradation or by inhibiting translation. Various approaches to research on miRNAs, target genes, and their regulatory mechanisms have emerged. The three most commonly used methods are computational predictions based on conserved sequences and secondary structures without experimental verification, the cloning of small RNA libraries, and direct capture of miRNAs by high-throughput sequencing^19^. Multiple experimental approaches were adopted to confirm the miRNA–mRNA target sites of plants, such as PPM-RACE, RLM-RACE, degradome sequencing, and Western blot^20,21^. Degradome sequencing is a combination of modified RLM-RACE and high-throughput deep sequencing and has been a powerful approach for large-scale miRNA target validation in plants.

Here, a genome of *O. javanica* cv. “Liyang Baiqin” was assembled. The evolution of the *O. javanica* genome and the contraction/amplification of gene families were investigated. Then, an online database of *O. javanica* was established. The sequences of the whole genome, nucleotides, and amino acids are available on Water dropwortDB. Some factors (such as genes and miRNAs) related to the phenotypic and anatomic divergence in *O. javanica* under different water conditions were further identified based on multi-omics association analysis. This study aimed to provide useful information on the water dropwort genome for the evolution and comparative genomics analyses and to identify the genetic factors related to the phenotypic and anatomic differences that emerged in *O. javanica* under different water conditions.

## Results

### Genome sequencing and assembly

Raw sequencing data were generated from the next-generation sequencing platform HiSeq 2000. In total, 32 libraries of six different insert sizes (180 bp, 200 bp, 500 bp, 800 bp, 2 kb, and 5 kb) were prepared. After filtering, a total of 175.40 Gb of clean data were obtained (Table 1). All clean data were assembled into contigs and scaffolds by using SOAPdenovo^22^. The resulting assembly spanned a 1.28 Gb genome that contained 213,461 contigs and 149,941 scaffolds (Table 2 and Supplementary Table 1). The N50 contig length was 13,040 bp, and the N50 scaffold length was 23,281 bp. Supplementary Table 2 provides genome information for several plants. The genome size of water dropwort was 3.03, 10.7, 3.42, and 3.2 times larger than that of *D. carota*, *A. thaliana*, *O. sativa*, and *P. trichocarpa*, respectively. The GC content of the water dropwort genome (32.97%) was similar to that of *D. carota* (34.8%), *A. thaliana* (36.06%), and *P. trichocarpa* (33.65%).

**Table 1:** Library information

Library insert size (bp)	Library number	Base number (Gb)
180	6	31.47
200	2	23.18
500	6	38.92
800	6	28.09
2000	8	25.86
5000	4	27.87
Total	32	175.40

**Table 2:** Genome assembly statistics

Feature	Value
Genome size (bp)	1,278,591,843
Genome GC %	32.97%
Gene number	42,270
Gene no. per 100 kb	3.31
Average gene length (bp)	3696.55
Exon region GC %	42.15%
Exon number	243,447
Average exon length (bp)	362.13
Exon no. per gene	5.76

### Gene prediction and annotation

**Fig. 2:**
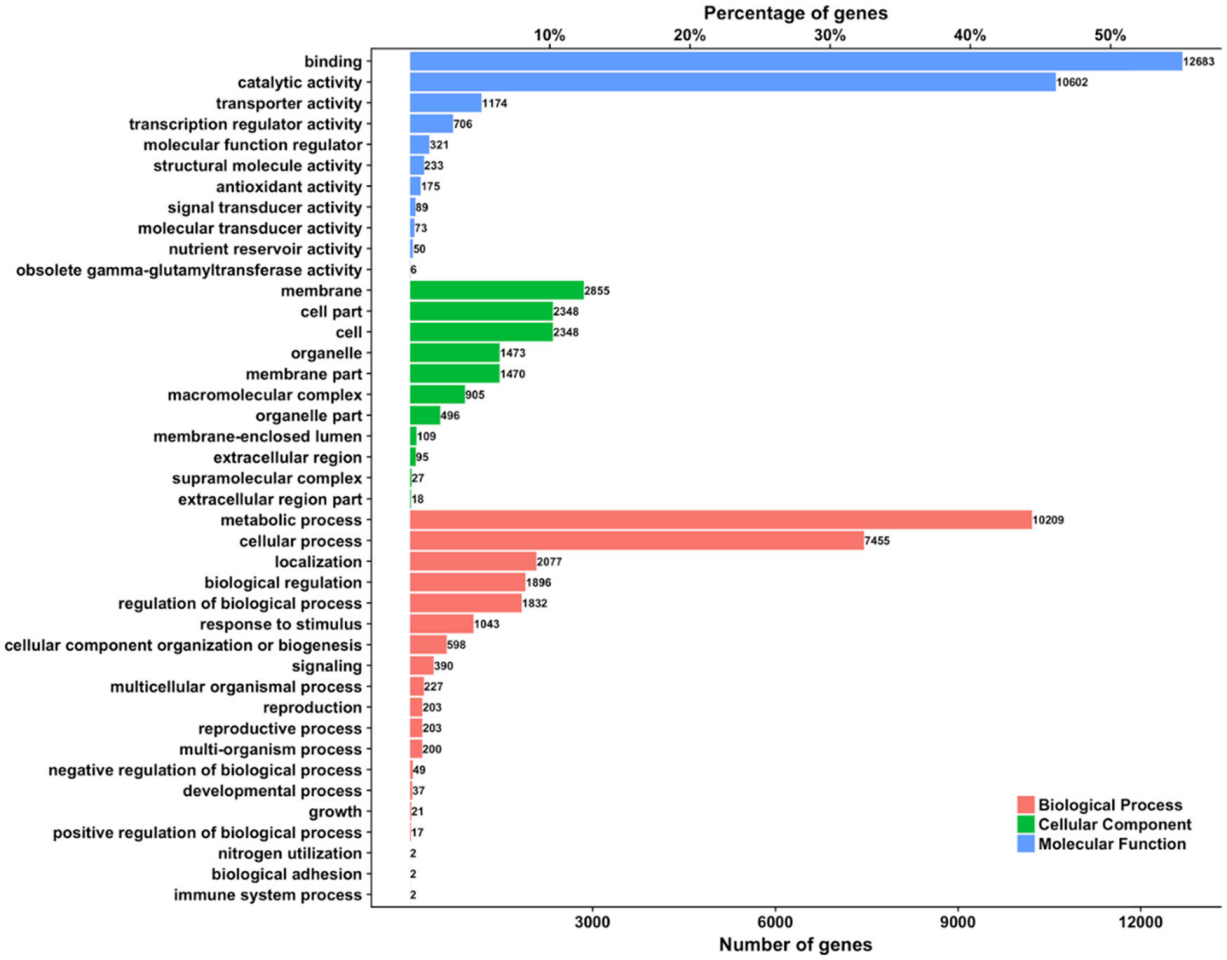
Distribution of Gene Ontology terms. The bar charts show the counts of genes involved in biological process (red), cellular component (green), and molecular function (blue) terms.

Ab initio and evidence-driven methods were applied to perform gene prediction. Functional annotation was accomplished using the non-redundant protein sequence databases UniProKB and InterPro. A total of 42,270 genes with an average length of 3,696.55 bp and 5.76 exons per gene were identified (Table 2). Among these genes, 39,699 genes could be functionally annotated, accounting for 93% of the whole genome (Supplementary Table 3). A total of 25,819 and 33,843 genes were annotated to the Nr and InterPro databases, respectively. A total of 22,999 genes were linked to the Gene Ontology (GO) terms across three classes: biological process terms (13,612), cellular component terms (4917), and molecular function terms (20,328). The gene distributions among these three classes are illustrated in Fig. 2. The annotation results showed that the majority of sequenced genes are involved in binding (55%) and catalytic (46%) activity. In terms of biological processes, the majority of genes were clustered in metabolic and cellular process terms (44% and 32% of all annotated genes, respectively). The majority of genes in the cellular component class contributed to the membrane, cell part, and cell terms (12%, 10%, and 10% of all annotated genes, respectively). Moreover, 319 miRNA, 1373 tRNA, 424 rRNA, and 786 snRNA genes were identified in the *O. javanica* genome (Supplementary Table 4).

### Clusters of orthologous genes

**Fig. 3:**
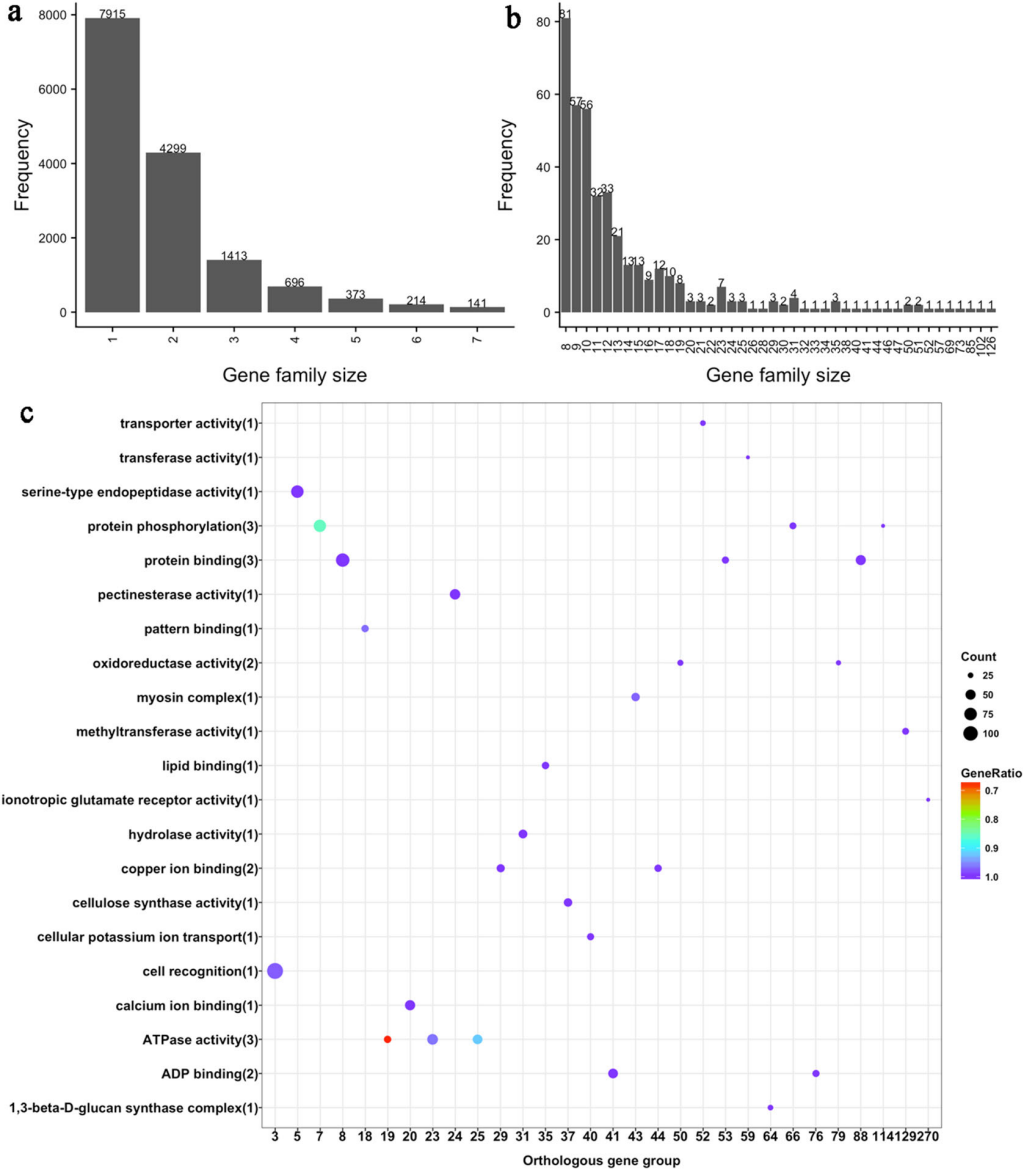
Frequency of gene family size and the top 30 large clusters of orthologous genes. **a** Frequency of gene families with a size greater than 100. **b** Frequency of gene families with a size less than 100. **c** The top 30 large clusters of orthologous genes. The number enclosed in parentheses is the number of orthologous gene groups annotated with the terms. Count: the number of genes related to the enriched GO term. GeneRatio: the ratio of genes related to the enriched GO term compared to the number of genes related to all GO terms in the cluster.

For the analysis of gene clusters, the OrthoMCL program was used to identify orthologous groups. A total of 33,497 genes were clustered into 15,451 orthologous groups with an average size of 2.17 genes (Supplementary Table 5). Only one gene was found in 7915 groups; thus, the size of these groups was one (Fig. 3a, b). The largest orthologous group was Group 3, containing 126 gene members. GO enrichment was performed for all gene members in the orthologous groups. The significantly enriched GO terms remained (adjusted *P* value < 0.05). Information on the top 30 large clusters of orthologous genes is listed in Fig. 3c. Most genes in the top 30 clusters only contributed to one GO term, except Groups 7, 19, and 25. The largest orthologous group also had the most gene members annotated to GO terms (122 genes).

### Analysis of gene family evolution

**Fig. 4:**
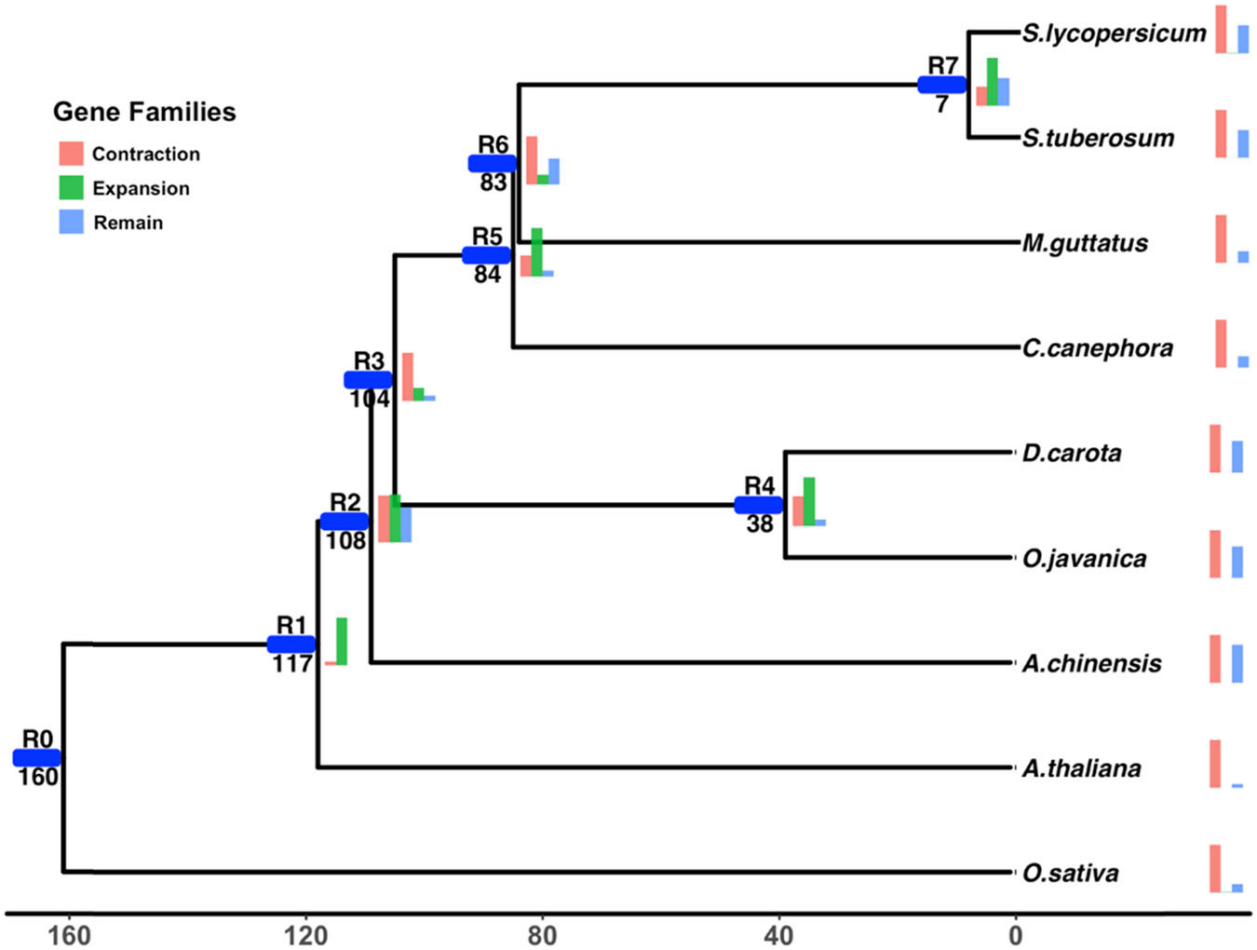
Genomic evolution and phylogenetic analyses. The blue line marks the divergence of branches. The text above the blue line records the divergence by order: R0-R7. The number under the blue line indicates the divergence time (unit: million years ago). The bar charts show the counts of expanded (green), contracted (red), and unchanged (blue) gene families in one branch compared to other branches.

A phylogenetic tree was constructed with the orthologous genes identified by OrthoMCL among nine plant genomes (*O. javanica*, *D. carota*, *Mimulus guttatus*, *Solanum tuberosum*, *Solanum lycopersicum*, *Oryza sativa*, *Coffea canephora*, *Actinidia chinensis*, and *Arabidopsis thaliana*) to analyze the differences in gene family size among taxa. The detailed changes are listed in Supplementary Table 6, and the data are shown as a bar plot in Fig. 4. *O. javanica* and *D. carota* diverged from each other approximately 38 million years ago. They both belong to the Apiaceae family, which diverged ~54 million years ago. *S. tuberosum* and *S. lycopersicum* belong to the Solanaceae family, which diverged approximately 7 million years ago, and have the smallest evolutionary distance. *M. guttatus*, *S. tuberosum*, *S. lycopersicum*, and *C. canephora* belong to euasterids I and have closer evolutionary distances. *O. javanica* and *D. carota* belong to euasterids II. *O. sativa* was located on a branch of monocots, exhibiting evolutionary divergence earlier than the others. The expansion and contraction of gene families in each branch are also shown on the phylogenetic tree. In *O. javanica*, 25 gene families were expanded, 17,179 gene families were contracted, and 11,322 gene families were not changed. In *D. carota*, 20 gene families were expanded, 17,196 gene families were contracted, and 11,352 gene families were not changed. In the branch containing both *O. javanica* and *D. carota*, 16,338 gene families were expanded, 10,068 gene families were contracted, and 2114 gene families were not changed.

### Water dropwortDB system

**Fig. 5:**
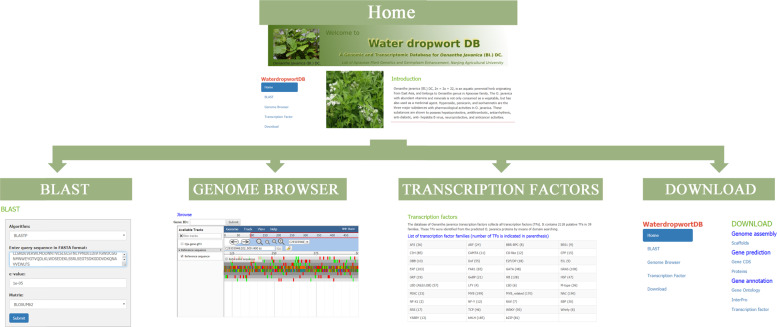
Organizational structure of the Water dropwortDB web pages. Home, BLAST, Genome Browser,Transcription Factor and Download interfaces composed of the organizational structure of Water dropwortDB.

To help researchers use our genomic data, an online database of the whole-genome sequences of water dropwort cv. “Liyang Baiqin”, Water dropwortDB, was constructed. The database is composed of five interfaces, including Home, BLAST, Genome Browser, Transcription factor (TF), and Download. An overview of the database architecture is shown in Fig. 5. Users can acquire basic information on the *O. javanica* genome online.

BLAST allows users to search the target genes with two BLAST search forms (BLASTP and BLASTX) based on sequence similarity. Users should enter the nucleotide or amino acid sequences in FASTA format in the frame and then set the suitable parameters to perform the search. When clicking the Submit icon, the web page will jump to the query results interface. A genome browser embedded in Water dropwortDB provides the detailed reference sequences of the *O. javanica* genome. The annotations of genes, mRNAs, coding sequences, and transcripts of each scaffold can be tracked with this tool. Users can obtain detailed information corresponding to the annotations of the selected scaffolds by clicking the different marked icons on this page.

In the Transcription factor section, 2118 putative transcription factors from 39 families in *O. javanica* are listed. In *O. javanica*, the majority of the transcription factors were clustered in the ERF (203), MYB (199), NAC (190), bHLH (185), MYB-related (170), HB (128), and GRAS (108) TF families. Users can download the nucleotide sequences and deduced amino acid sequences of all the putative TF genes in the Download section. In addition to the online search tool for obtaining target gene information, access to download the genome data has been provided. The assembled scaffolds, the CDSs of predicted genes, the amino acid sequences of predicted proteins, and the gene annotations (based on GO, InterPro, and Nr databases) of the *O. javanica* genome are available when users log in.

### Morphological and anatomic characteristics of *O. javanica* under different water conditions

**Fig. 6:**
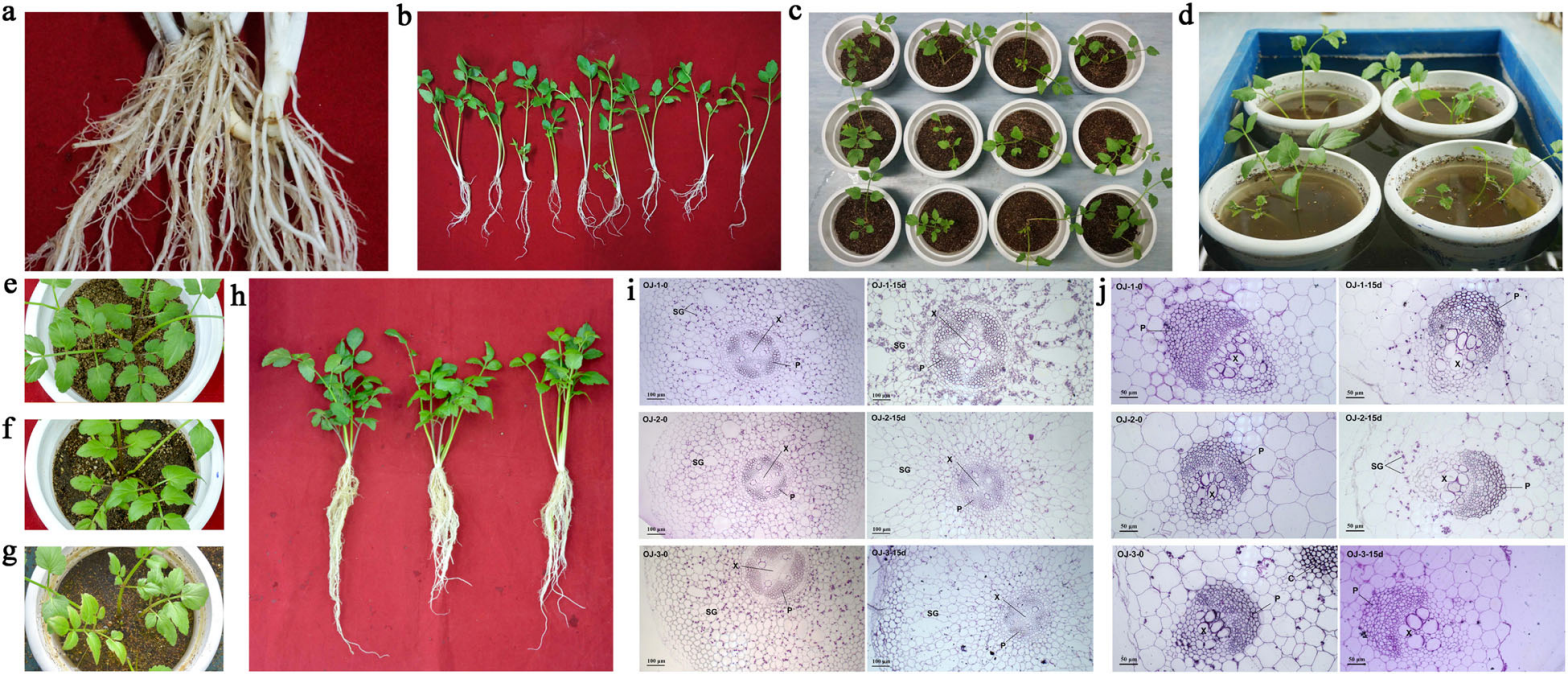
Plant growth and anatomical structure of *O. javanica* under different water conditions. **a** Plants on one stolon. **b** Plants of a similar size divided from one stolon. **c** Plants planted in pots. **d** The plants and their pots were immersed in water that was 4 cm higher than the soil in the pots. **e** Soil with deficient water (OJ-1). **f** Soil with sufficient water (OJ-2). **g** Soil with excess water (OJ-3). **h** Plant phenotypes under water-deficient (OJ-1), water-sufficient (OJ-2), and waterlogging conditions (OJ-3) successively from left to right. **i** Root structure of *O. javanica* under different water conditions. SG starch grain, P phloem, X xylem. **j** Petiole structure of *O. javanica* under different water conditions. SG starch grain, P phloem, X xylem

Fresh plants divided from stolons of similar size were treated with different water conditions (Fig. 6a–d). The seedling state after treatment is shown in Fig. 6e–h. After 15 days of treatment, two new petioles emerged in all lines. The two petioles of OJ-1 turned purple, the leaves were green, and the roots became more fibrous. For OJ-2 plants, the two petioles turned light purple. For OJ-3 plants, the petioles remained green, the part above the ground looked healthier than that in other treatments, and the leaves looked fresh and tender with a high water content (Fig. 6h).

The root and petiole structural divergence of *O. javanica* under different water conditions was detected using resin-embedding microtomy and histochemistry staining. As shown in Fig. 6i, after 15 days of treatment, the xylem of the OJ-1 roots grew rapidly, and starch grains increased. For the roots of OJ-2 and OJ-3, xylem growth was not obvious, and the starch grains had no remarkable increase. As shown in Fig. 6j, after 15 days of treatment, no remarkable anatomic difference occurred during petiole growth.

### Transcriptome and microRNA sequencing of *O. javanica* under different water conditions

Transcriptome sequencing resulted in 4.25, 4.03, and 4.02 Gb of raw data from OJ-1, OJ-2, and OJ-3 (Supplementary Table 7). For all three samples, the Q20 values were greater than 90, and the Q30 values were greater than 85, indicating that more than 90% of the raw data were within a sequencing error rate of 1%, and more than 85% of the raw data were within a sequencing error rate of 0.1%. By calculating the percentage of G and C bases out of the total nucleotides, the GC values were found to be approximately equal to 45%.

**Fig. 7:**
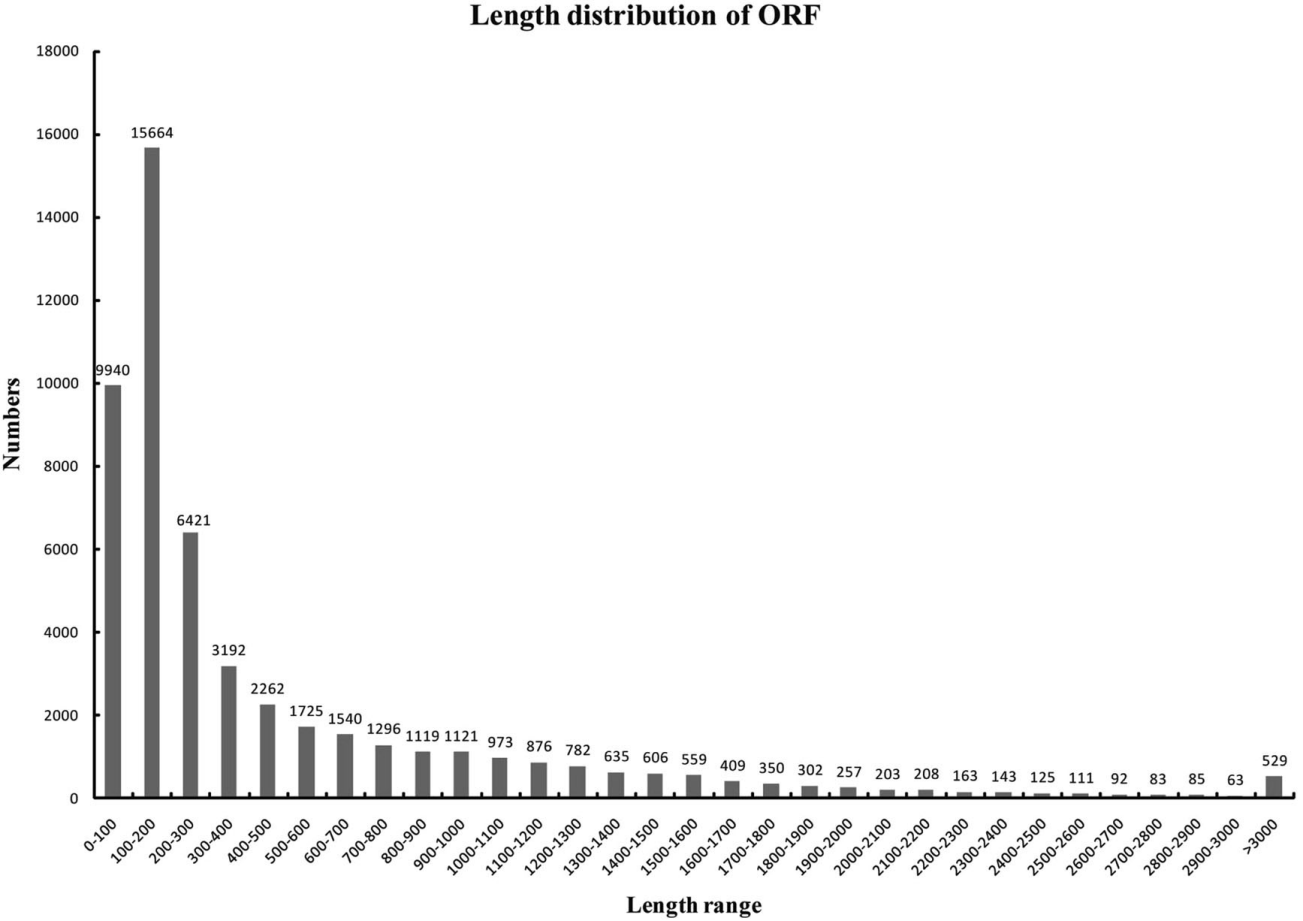
Length distribution of the open-reading frames (ORFs). The figures on the column chart represent the counts of ORFs distributed within the corresponding length range.

De novo transcriptome assembly was performed using Trinity on the basis of the sequencing data from all three samples^23^. An overview of the assembled transcriptome data is shown in Supplementary Table 8. Most of the contigs, 6,815,831 (99.42%), had a size of 200–300 bp. Finally, all short sequences were assembled into 102,055 transcripts and 52,083 unigenes. The average length of the unigenes was 769.62 bp, and the median length was 1393 bp. The open-reading frame (ORF) was predicted using Getorf software, and the length distribution of the ORFs is shown in Fig. 7. A total of 6801 unigenes (13.12%) with a length distribution ranging from 501 to 1000 bp and 7554 unigenes (14.57%) with a length of more than 1000 bp were found in the ORF prediction.

Small RNA sequencing produced 20.53, 20.29, and 18.36 Mb of raw data for OJ-1, OJ-2, and OJ-3 (Supplementary Table 9). A total of 14.98, 14.27, and 13.79 Mb clean reads were obtained from OJ-1, OJ-2, and OJ-3 after filtering out the low-quality reads, trimming the adapters, and removing the overrepresented sequences and noise.

### Analysis of differentially expressed genes (DEGs) in *O. javanica* under different water conditions

**Fig. 8:**
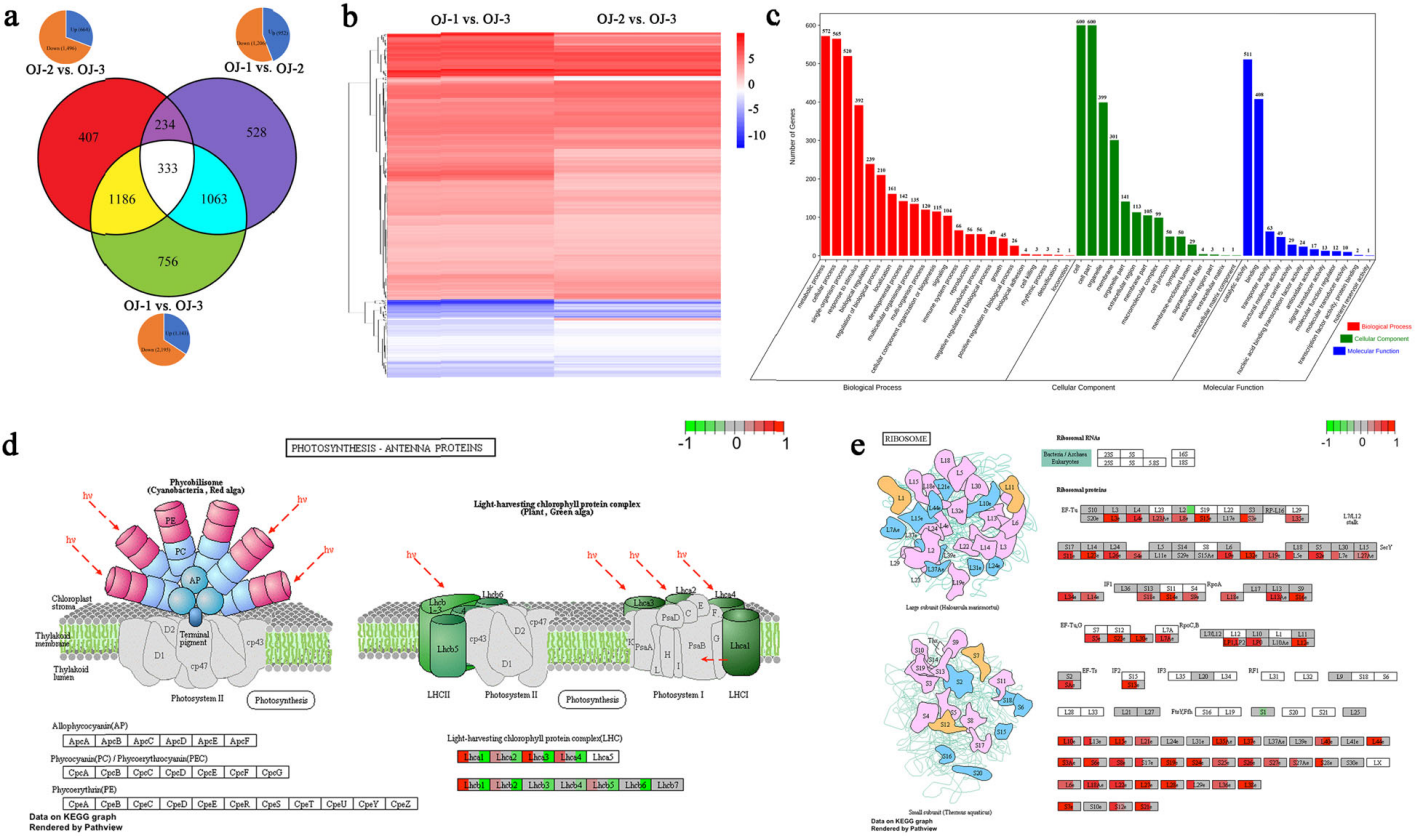
Analysis of differentially expressed genes in *O. javanica*. **a** Statistics of differentially expressed genes in *O. javanica* under different water conditions. OJ-1: plant under water-deficient conditions; OJ-2: plant under water-sufficient conditions; and OJ-3: plant under waterlogging conditions. **b** Hierarchical clustering of 1519 DEGs in OJ-1 and OJ-2 compared to OJ-3. OJ-1 vs. OJ-3: log of RPKM_(OJ−1)_/RPKM_(OJ−3)_; OJ-2 vs. OJ-3: log of RPKM_(OJ−2)_/RPKM_(OJ−3)_. **c** GO enrichment analysis of 1519 DEGs. **d** Differentially expressed genes of *O. javanica* in the photosynthesis pathway under different water conditions. Colors represent three sets of data, from left to right: OJ-3 vs. OJ-1, OJ-3 vs. OJ-2, OJ-1 vs. OJ-2. **e** Differentially expressed genes of *O. javanica* in the ribosome pathway under different water conditions. Colors represent three sets of data, from left to right: OJ-3 vs. OJ-1, OJ-3 vs. OJ-2, OJ-1 vs. OJ-2.

In total, 4507 DEGs were identified in *O. javanica* under three different water conditions (Fig. 8a). As an aquatic vegetable, waterlogging conditions (OJ-3) are more conducive to the growth of *O. javanica*, whereas water-deficient conditions (OJ-1) and water-sufficient conditions (OJ-2) are relatively dry conditions. Upon selecting OJ-3 as the control, a total of 1519 DEGs were identified in OJ-1 vs. OJ-3 and OJ-2 vs. OJ-3. The expression data and functional annotations are listed in Supplementary Table 10. Compared with OJ-3, OJ-1 showed 1143 upregulated and 2195 downregulated genes, while 664 and 1496 genes were upregulated and downregulated in OJ-2, respectively. Based on the DEGs shown in Fig. 8b, the expression patterns of 1519 DEGs in OJ-1 vs. OJ-3 and OJ-2 vs. OJ-3 were similar. Then, the DEGs were classified into different functional categories by GO enrichment analysis (Fig. 8c and Supplementary Table 11). In the biological process category, the metabolic process (71.23%) was the most abundant GO term. Some of the important biological functions included anatomical structure morphogenesis (61 genes, 7.60%), response to water deprivation (35 genes, 4.36%), root system development (32 genes, 3.99%), cellular response to stress (23 genes, 2.86%), water transport (11 genes, 1.37%), photosynthesis (4 genes, 0.5%), and cellular response to water deprivation (3 genes, 0.37%). Four DEGs were associated with photosynthesis, including downregulated *Oja34996* (annotated as ribulose bisphosphate carboxylase/oxygenase activase) and *Oja40226* and upregulated *Oja37779* (annotated as NAD(P)H-quinone oxidoreductase subunit K) and *Oja40814* (annotated as geranylgeranyl diphosphate reductase). *Oja17217* (annotated as multiprotein-bridging factor 1), *Oja16558* (annotated as protein phosphatase 2 C), and *Oja25141* (annotated as glutathione S-transferase 1) were found to be involved in the cellular response to water deprivation; the former was downregulated in OJ-1 and OJ-2 compared with OJ-3, whereas the latter two were upregulated. For the cellular component category, cell (85.47%) and cell part (85.47%) were the most highly represented groups. In the molecular function category, catalytic activity (68.41%) and binding (54.62%) represented the top two categories.

In addition, 333 genes were differentially expressed among OJ-1, OJ-2, and OJ-3 (Supplementary Table 12). Hierarchical clustering of 333 DEGs is shown in Supplementary Fig. 1a. GO enrichment analysis showed that the functional categories of these 333 DEGs shared a similar pattern with those of the whole set of 1519 DEGs (Supplementary Fig. 1b). Metabolic process (122 genes), cell (134 genes), and catalytic activity (113 genes) were represented at the highest scale in the categories of biological process, cellular component, and molecular function, respectively. *Oja34996* (annotated as ribulose bisphosphate carboxylase/oxygenase activase) and *Oja40814* (annotated as geranylgeranyl diphosphate reductase), which are associated with photosynthesis, were also present in the 333 DEGs. *Oja16558* (annotated as protein phosphatase 2C), which is related to the cellular response to water deprivation, was also identified. The 333 DEGs were further clustered into 14 groups for functional annotation, as shown in Supplementary Table 13. The majority of DEGs shared the same regulation profiles in OJ-1 vs. OJ-3, OJ-2 vs. OJ-3, and OJ-1 vs. OJ-2. Many genes were regulated more intensely in OJ-1 than in OJ-2 or OJ-3, except for Groups 2, 11, and 12. We examined the functional annotation of the genes in those groups (in addition to those in Groups 2, 7, 8, 11, and 12). The following interesting functionally annotated genes were found: glucan water dikinase (GWD)-related genes, which function in starch metabolism^24^; laccase-related genes, which function in lignin biodegradation; anthocyanin regulatory C1 protein-related genes; dehydrin COR47- and DHN1-related genes, which were reported to be strongly induced by drought^25,26^; hormone-related genes, which are very important for the growth of plants^27^; and aquaporin-related genes, which play an important role in the regulation of plant-water balance^28^.

Mapping of the DEGs to the KEGG pathway showed that many genes in the same pathway had a similar gene expression trend. For example, in the photosynthesis pathway, most of the *LHCP* genes in OJ-1 were expressed at lower levels than those in OJ-2 and OJ-3. Moreover, no significant differences were found between the genes in OJ-2 and OJ-3 (Fig. 8d). In the ribosome pathway, the expression of most genes in OJ-3 was significantly higher than that in OJ-1 and OJ-2, while the gene expression differences between OJ-1 and OJ-2 were not remarkable (Fig. 8e).

### Analysis of differentially expressed miRNAs and potential miRNA targets in *O. javanica* under different water conditions

**Fig. 9:**
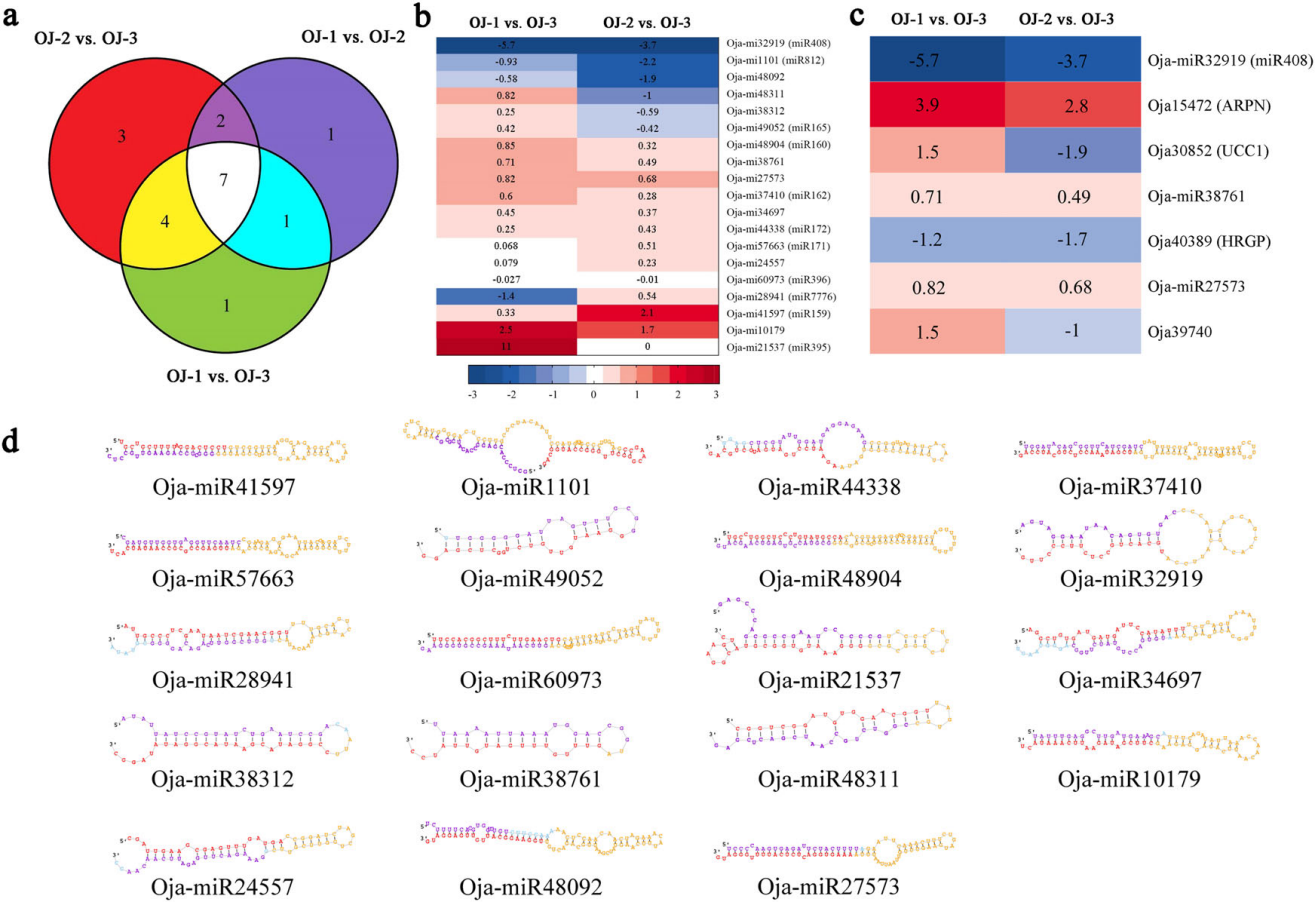
Analysis of differentially expressed miRNAs in *O*. javanica. **a** Statistics of differentially expressed miRNAs in *O. javanica* under different water conditions. **b** Heatmap of differentially expressed miRNAs. The names of the conserved miRNAs are shown in brackets. OJ-1 vs. OJ-3: log of RPKM_(OJ−1)_/RPKM_(OJ−3)_; OJ-2 vs. OJ-3: log of RPKM_(OJ−2)_/RPKM_(OJ−3)_. **c** Heatmap of miRNAs and their corresponding potential target genes. The corresponding target genes for each miRNA are shown. The name of the conserved miRNA or target gene symbol is shown in brackets. OJ-1 vs. OJ-3: log of RPKM_(OJ−1)_/RPKM_(OJ−3)_; OJ-2 vs. OJ-3: log of RPKM_(OJ−2)_/RPKM_(OJ−3)_. **d** The secondary structure prediction of the precursors of differentially expressed miRNAs. Red represents the mature sequence; yellow represents the ring structure; and purple represents the star sequence.

Analysis of miRNA expression in *O. javanica* under different water conditions revealed that 19 miRNAs were differentially expressed (Fig. 9a). Among them, seven miRNAs were differentially expressed under all three water conditions. All 19 differentially expressed miRNAs are shown in Fig. 9b.

The complementary mRNA sequences were searched in the corresponding transcriptome sequence data to predict the potential targets of the miRNAs. A total of 46 potential targets were predicted for the 19 differentially expressed miRNAs. They were annotated as scarecrow-like protein, growth-regulating factor 4, E3 ubiquitin-protein ligase UPL2-like, disease-resistance protein RPS2, and others (Supplementary Table 14). Among the 46 potential target genes, four genes were found to be differentially expressed under different water conditions (Fig. 9c). Oja-miR32919 (miR408) was negatively regulated in OJ-1 and OJ-2 compared with OJ-3, while its corresponding target gene *Oja15472* (*ARPN*, plantacyanin) was positively regulated. Moreover, the regulation of Oja-miR32919 and *Oja15472* was more intense in OJ-1 than in OJ-2. Both *Oja30852* (*UCC1*, uclacyanin 1) and *Oja40389* (*HRGP*, hydroxyproline-rich glycoprotein) showed negative regulation in OJ-2 vs. OJ-3.

The structural features of the miRNA precursors are shown in Fig. 9d. The precursors of differentially expressed miRNAs contained a typical hairpin structure. miRNA and miRNA stars were produced by two arms of the same hairpin structure. Precursor miRNAs (pre-miRNAs) were transported out of the nucleus with the help of exportin-5. They were digested by the cytoplasmic Dicer enzyme and became mature miRNAs. miRNA stars were usually degraded rapidly after the mature body formed.

### Degradome sequencing and data summary

**Fig. 10:**
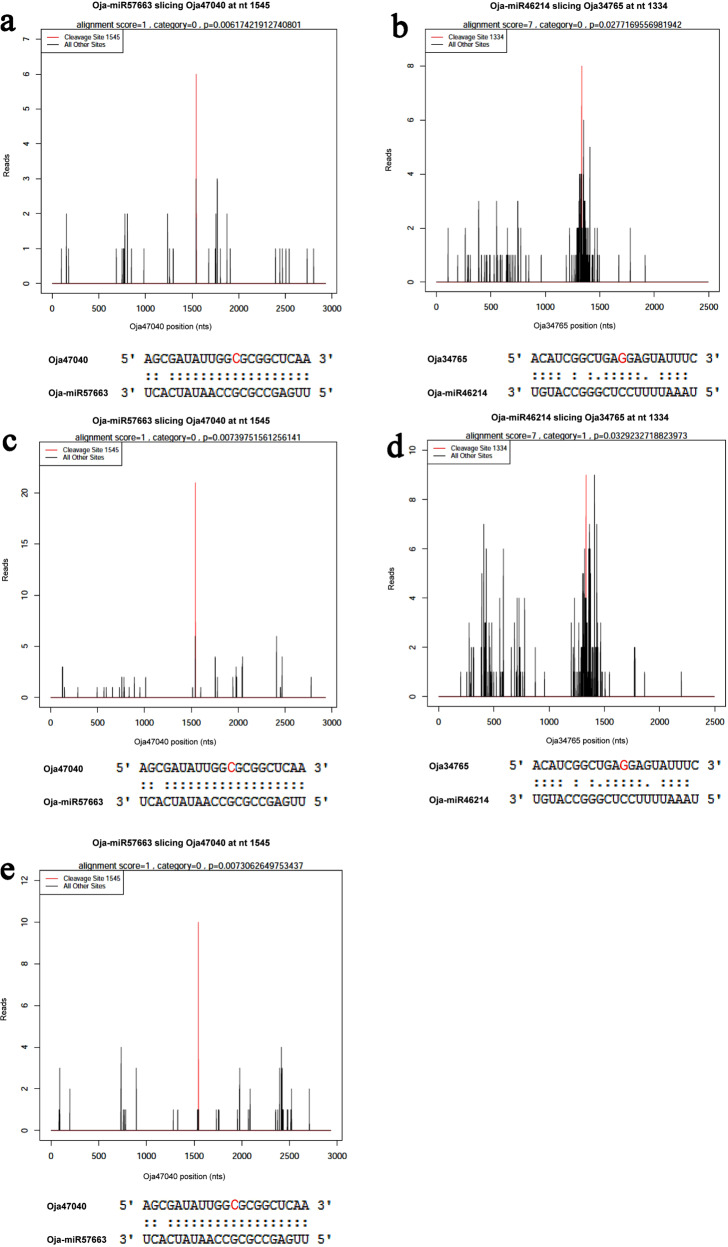
Target plots (t-plots) of miRNA targets confirmed by degradome sequencing. **a**, **b** were found in OJ-1 (water-deficient condition); **c**, **d** were found in OJ-2 (water-sufficient condition); and **e** was found in OJ-3 (waterlogging condition).

Three degradome libraries based on captured cleaved mRNAs were constructed from the petioles of *O. javanica* under three different water conditions. In higher plants, most miRNAs regulate their targets via cleavage, which normally occurs between the 10th and 11th nucleotides of the complementary region between the miRNA and the mRNA target^21,29^. A total of 14 pairs of miRNAs and targets were captured at the predicted cleavage site by degradome analysis (Table 3). Oja-miR57663 targeting *Oja47040* was found in all three degradome libraries (Fig. 10a, c, e), and the cleavage tag was the most abundant degradome sequence matching the target. Oja-miR57663 was perfectly matched to ath-miR171b (miR171). Oja47040 was annotated as a scarecrow-like protein (SCL6/22/27), which was reported to be a miR171-targeted protein and to negatively regulate chlorophyll biosynthesis^30^. No remarkably different expression patterns of *Oja47040* were found among OJ-1, OJ-2, and OJ-3. Oja-miR46214 targeting *Oja34765* was found in OJ-1 and OJ-2 (Fig. 10b, d). In OJ-1, this cleavage tag was the most abundant degradome sequence matching the target. Oja-miR46214 was perfectly matched to lja-miR7519.

**Table 3:** Identified miRNA targets found by degradome sequencing.

Tissue	miRNA	Conserved miRNAs	Target	Target annotation	C.Site	Category	*P* value
OJ-1	Oja-miR57663*	ath-miR171b	*Oja47040*	Scarecrow-like protein	1545	0	0.0062
	Oja-miR46076	bdi-miR7764	*Oja50133*	MuDR family transposase	351	0	0.0231
	Oja-miR46214	lja-miR7519	*Oja30143*	Lipid-transfer protein DIR1	542	1	0.0418
	Oja-miR46214*	lja-miR7519	*Oja34765*	DDE	1334	0	0.0277
OJ-2	Oja-miR26176	unconservative	*Oja46178*	Uncharacterized protein	150	2	0.0473
	Oja-miR60973	ptc-miR396f	*Oja40509*	Strictosidine synthase 3	1301	1	0.0490
	Oja-miR57663	ath-miR171b	*Oja44595*	PK superfamily	904	0	0.0142
	Oja-miR35317	unconservative	*Oja33469*	No annotation	196	3	0.0169
	Oja-miR57663*	ath-miR171b	*Oja47040*	Scarecrow-like protein	1545	0	0.0074
	Oja-miR49052	ath-miR165a	*Oja46154*	Presequence protease 2	870	0	0.0345
	Oja-miR46214*	lja-miR7519	*Oja34765*	DDE	1334	1	0.0329
OJ-3	Oja-miR46056	ath-miR8173	*Oja44290*	UDP-glucuronate 4-epimerase 6	1420	3	0.0380
	Oja-miR60973	ptc-miR396f	*Oja38638*	Uncharacterized protein	290	0	0.0403
	Oja-miR45727	ath-miR8167a	*Oja44795*	Pentatricopeptide	1540	4	0.0430
	Oja-miR38312	unconservative	*Oja33941*	Uncharacterized protein	381	4	0.0324
	Oja-miR57663*	ath-miR171b	*Oja47040*	Scarecrow-like protein	1545	0	0.0073
	Oja-miR35317	unconservative	*Oja39892*	Transcription factor	700	1	0.0104

^*^are miRNAs whose targets are shown in the target plots (t-plots) (Fig. 10).

## Discussion

Omics includes genomics, transcriptomics, proteomics, RNomics, and others. With the continuous development of sequencing technology and sequencing methods, multi-omics association analysis has been widely used to reveal the molecular regulatory mechanisms in plants responding to environmental change^31^. First, whole-genome sequencing, an omics technique, can quickly obtain a large amount of biological information for the exploration of gene structures, the establishment of gene maps, and mining of gene functions. *O. javanica* is an important aquatic perennial vegetable crop from the Apiaceae^5,6^. It has strong adaptability to waterlogging resistance. *O. javanica* is well known for its abundant nutrition and pharmacological value, and the main method of production is vegetative propagation. At present, several reports on the cultivation techniques, nutrients, and medicinal components of *O. javanica* have been carried out. Few studies on the molecular biology of *O. javanica* have been performed, and the lack of genomic information limits further research. The number, structure, and function of genes change continuously due to the evolution of a species, the formation of physiological mechanisms, and adaptation to different living environments^32^. Different genes arose from the divergence of species or from whole-genome duplication events within species. Genome sequencing and comparative genome analysis of *O. javanica* are important for understanding the underlying evolution mechanism and biological functions of *O. javanica*.

In this study, the whole-genome sequences of *O. javanica* cv. “Liyang Baiqin” were obtained by high-throughput sequencing, and an overall sequence length of 1.28 Gb was assembled. The average contig N50 length was 13,040 bp, while the scaffold N50 length was 23,281 bp. In Apiaceae, the genome sizes of carrot, coriander, and celery were 421.5 Mb, 2118.31 Mb, and 3.33 Gb^33–35^, respectively. A total of 42,270 genes were identified in the genome of *O. javanica*, of which 93.92% of genes were functionally annotated. Computational Analysis of gene Family Evolution (CAFE) software with a stochastic birth and death process was used to model the gene family evolution across the phylogenetic tree^36,37^. The distribution of gene family size showed significant changes in family size (expansion or contraction) throughout phylogenetic history. The origin, evolution, and genetic relationship of *O. javanica* were revealed from the phylogenetic tree. Apiaceae appeared on Earth 54 million years ago. *O. javanica* is a close relative of *D. carota*, and the divergence of the two species occurred approximately 38 million years ago. Combining gene family amplification/contraction with gene function annotation could help identify genetic information related to species-specific characteristics and further solve the biological problems associated with those characteristics, such as the waterlogging tolerance mechanism of *O. javanica*.

On the basis of the completion of the whole-genome sequence of *O. javanica*, a genomic database (Water dropwortDB) was constructed for use by the global scientific community. Water dropwortDB contains five interfaces (Home, BLAST, Genome Browser, TF, and Download). The interface Water dropwortDB is user-friendly and publicly available (http://apiaceae.njau.edu.cn/waterdropwortdb). With the development of sequencing technology, we will continuously collect more genome and transcriptome data to update the current database^38–40^. Water dropwortDB could be a powerful tool to further research on the molecular biology of water dropwort.

*O. javanica* is a healthy vegetable that is generally planted in wet soils or water. This study focused on the effects induced by different water conditions on *O. javanica* and attempted to identify some factors (genes and miRNAs) related to the apparent and anatomic differences that emerged in *O. javanica* under three water conditions. After 15 days of water treatment, OJ-3 had a better appearance than OJ-1 and OJ-2. Regarding anatomy, the xylem transported water and soluble mineral nutrients from the roots throughout the plant. The root xylem grew rapidly and starch grains increased in OJ-1. Among the water-deficient and waterlogging conditions, a large number of DEGs involved in the stress response, photosynthesis, and anatomical structure morphogenesis were identified, indicating that the physiological changes caused by water deficiency were closely related to the regulation of DEGs. A total of 61 and 23 DEGs were associated with anatomical structure morphogenesis and cellular response to stress, respectively. The DEGs that encoded ribulose bisphosphate carboxylase/oxygenase activase, NAD(P)H-quinone oxidoreductase subunit K and geranylgeranyl diphosphate reductase were involved in photosynthetic regulation. Three DEGs (*Oja17217*, *Oja16558,* and *Oja25141*) play potentially important roles in the cellular response to water deprivation. These genes encode GWD, which is involved in the phosphorylation of amylopectin, an essential step within starch metabolism^24^. This phenomenon could be associated with the apparent increase in starch grains in OJ-1. Aerobic conditions are important for laccase, a glycoenzyme involved in lignin biodegradation^41^. This phenomenon could explain why the xylem in OJ-1 grew rapidly. In addition, genes that encode the dehydrins COR47 and DHN1 were identified. They belong to the acidic dehydrin family, and they were reported to be strongly induced by low temperature and drought^25,26^. Two aquaporin genes were found in Group 9. Aquaporin plays an important role in the regulation of plant-water balance, growth regulation, and response to abiotic stress factors due to its involvement in the transport of water and other small solutes across cell membranes. It also plays a crucial role in osmotic water fluxes during drought and recovery^28,42^. Further work is planned on these genes in future research.

Mapping the DEGs to KEGG pathways revealed that the expression levels of genes in the same pathway were similar. In the photosynthesis pathway, the expression of most of the *LHCP* genes in OJ-1 was significantly lower than that in OJ-2 and OJ-3. This finding is consistent with the expression trend of the *Lhcb1* gene under drought stress found in previous research on *O. javanica*^13^. This finding indicated that photosynthesis weakens when *O. javanica* is under drought stress. In the ribosome pathway, the expression of most genes in OJ-3 was significantly higher than that in OJ-1 and OJ-2. Ribosomes are in charge of the translation process of mRNA to protein in all cells, and they are essential organelles for cell growth^43,44^. In the case of *O. javanica* under waterlogging conditions, ribosome-related genes were more highly expressed than other genes. This finding showed that waterlogging is the most suitable condition for the growth of *O. javanica*, with its protein translation process being the most active.

miRNAs are essential regulatory factors involved in plant growth and adaptation to stress. The precursors of miRNAs contain a typical hairpin structure^45^. miRNA and miRNA stars are produced by two arms of the same hairpin structure. Nineteen miRNAs were found to be differentially expressed under different water conditions. The miRNA Oja-miR32919 was downregulated in OJ-1 and OJ-2 compared with that in OJ-3, while its corresponding target gene *Oja15472* (*ARPN*) was upregulated. Oja-miR32919 was matched to miR408, which was reported to be downregulated by water deficit^46^, and miR408 overexpression enhanced drought tolerance^47^. *Oja15472* (*ARPN*) was related to basic blue protein according to BLAST. In this study, *Oja15472* (*ARPN*) was found to be regulated by miR408 and downregulated by water deficit. Degradome analysis showed that 14 pairs of miRNAs and targets were captured at the predicted cleavage site, thus providing experimental evidence to support the previous computational predictions. Oja-miR57663 (perfectly matched to ath-miR171b) targeting *Oja47040* (annotated as a scarecrow-like protein belonging to the GRAS gene family) was found in all three degradome libraries. This finding is similar to that of previous research, which revealed that miR171 targeted genes that encoded scarecrow-like proteins (SCL6/22/27)^30,48^. However, no remarkably different expression profiles of *Oja47040* were found among the plants under different water conditions. More precise experiments are needed in the future to reveal the regulatory mechanism between Oja-miR57663 (miR171) and *Oja47040* and how they are related to chlorophyll accumulation and branch development in plants. The results provide a foundation for understanding how *O. javanica* responds to water stress, including morphological, anatomical, and genetic differentiation.

## Materials and methods

### Material preparation, genome sequencing, and assembly

The plant material used in this study was an *O. javanica* cultivar called “Liyang Baiqin”, which was derived from the Li Yang region, Jiangsu Province, China. *O. javanica* extended the range of its community through the perpetual clonal growth of stolons. We obtained water dropwort resources and planted them in the State Key Laboratory of Crop Genetics and Germplasm Enhancement, Nanjing Agricultural University, China.

The genomic DNA used for genome sequencing was extracted from young leaves of “Liyang Baiqin” via a modified CTAB method^49^. Genome sequencing was performed by Beijing Genomics Institute-Shenzhen (BGI-Shenzhen) on an Illumina HiSeq 2000 (San Diego, CA, USA). Six different insert sizes (180 bp, 200 bp, 500 bp, 800 bp, 2 kb, and 5 kb) were applied to prepare the libraries. Adapters and low-quality reads were removed from the raw data by using CutAdapt. Paired-end reads from the short-insert-size libraries (180, 200, 500, and 800 bp) were used to assemble the genome into contigs using SOAPdenovo2 (http://soap.genomics.org.cn/soapdenovo.html)^22^ with k-mer = 63. Then, all sequence data were aligned to these contigs, and scaffolds with mate-pair information were generated based on the estimated insert size (from 180 bp to 5 kb). The gaps caused by scaffolds were closed by GapCloser. The assembled genome was deposited in GenBank under the accession number QRFB00000000, and the version described in this paper was QRFB01000000.

### Gene prediction and annotation

The protein sequences from six plants (*A. thaliana*, *M. guttatus*, *S. tuberosum*, *S. lycopersicum*, *D. carota*, and *O. sativa*) were downloaded from Phytozome (https://phytozome.jgi.doe.gov) to predict the protein-coding genes in the *O. javanica* genome. MAKER^50^ was used to convert the genomic sequence into a genome database. Protein sequences were aligned to the genome, and then ab initio and evidence-driven gene predictions were generated.

SNAP^51^ and Augustus v3.2.2^52^ were adopted for the ab initio gene prediction. For evidence-driven gene prediction, MAKER used four external programs: RepeatMasker (http://repeatmasker.org), BLAST, Exonerate v2.2.0^53^, and SNAP^51^. RepeatMasker and BLAST were used to identify and mask repeats. BLAST is rapid, but it has no model for splice sites. Therefore, Exonerate v2.2.0^53^, a splice-site-aware alignment algorithm, was used to realign and polish sequences after filtering and clustering to improve information about splice sites and exon boundaries^54^. SNAP was used to synthesize information from the polished and clustered protein alignments to identify the most representative prediction^51^. For quality control, a metric called Annotation Edit Distance (AED)^55^ was employed. Finally, non-redundant proteins with the best intron-exon structures were obtained from the overlapping predictions.

The predicted protein-coding genes were annotated by the non-redundant protein sequence database UniProKB (http://www.uniprot.org) using BLASTp. The annotation information with the best BLAST hit against the database was chosen. Motifs and protein domains were scanned by InterProScan v5^56^ against the InterPro^57^ signatures, including Pfam^58^, PRINTS^59^, and SMART^60^. After searching was performed, the corresponding InterPro entries and GO^61^ terms were obtained for each gene.

### Identification of orthologous groups and transcription factors

The OrthoMCL^62^ program was used to identify the orthologous groups with the Markov cluster algorithm (MCL). The procedure started by performing all-against-all BLASTP comparisons with the proteins of *O. javanica*, *D. carota*, *S. tuberosum*, *S. lycopersicum*, *O. sativa*, and *A. thaliana*. BLAST results with an *E* value <1 × 10^−5^ were retained. The inflation value is an important parameter of the MCL algorithm. A lower inflation value indicates looser clusters, which could result in more sequences being clustered into fewer groups^62^. An inflation value of 1.5 was applied in this study. The transcription factors in the *O. javanica* genome were identified according to PlnTFDB^63^_._

### Computational analysis of gene family evolution

The dynamic evolution process of gene families was constructed using CAFÉ version 3.1 with a probabilistic graphical model^35^. The evolutionary timescale was directly retrieved from http://www.timetree.org^64^ and used to construct a primary phylogenetic tree. Redundant sequences (≥ 90% identity) from the same organism were removed using CD-HIT^65^. Orthologous clusters were identified among the nine plant genomes (*O. javanica*, *D. carota*, *M. guttatus*, *S. tuberosum*, *S. lycopersicum*, *O. sativa*, *C. canephora*, *A. chinensis*, and *A. thaliana*) by OrthoMCL. Then, multiple alignments were performed for each cluster by using MUSCLE version 3.8.31^66^ with default parameters. The clusters containing only one copy in only one organism were removed from further analysis. The multiple alignments in clusters were then concatenated into gene families. FastTree^67^ was employed to reconstruct a maximum likelihood phylogenetic tree for these gene families.

### Database construction

The collective data presented in this database are expected to provide valuable resources for genetic and genomic studies on *O. javanica*. An Apache HTTP server in a Linux (CentOS6.2) operating system was used for the web development of Water dropwortDB. PHP5, Perl scripts, HTML and JavaScript were also used to construct the website. The database was installed with the BLAST and Generic Genome Browser (GBrowse1.7) packages^68^, thus allowing users to access BLAST and browse the water dropwort genome data online. The interface of Water dropwortDB is user-friendly and easy to operate.

### Water stress treatments of *O. javanica*

The germplasm resources of *O. javanica* cv. “Liyang Baiqin” were deposited at the State Key Laboratory of Crop Genetics and Germplasm Enhancement, Nanjing Agricultural University (32°04′N, 118°85′E). *O. javanica* extended the range of its community through the perpetual clonal growth of stolons. Small fresh plants were divided from the stolon. Plants of a similar size were selected and planted in pots containing a 1:1 soil/vermiculite mixture in a controlled environment growth chamber programmed for a 12/12 h light and dark cycle at 22 °C/18 °C (day/night). One week after planting the clonal ramet, the plants were randomly divided into three groups with different water treatments: OJ-1 (the original water in pots was drained, and no water irrigation was performed in the following days), OJ-2 (water irrigation was performed two times every day, and the soil was kept under water-sufficient conditions), and OJ-3 (water was kept 4 cm higher than the soil in the pots). After 15 days, the petioles and roots were collected and stored for anatomical observation and high-throughput sequencing.

### Resin-embedded microtomy

Plant samples under different water conditions were cut from healthy plants by using a razor blade. Resin-embedded sections were used to prepare *O. javanica* samples for anatomic observation in accordance with a previous method^69,70^.

### RNA isolation, library preparation, sequencing, and analysis for transcriptome sequencing

Total RNA was isolated from the petioles of OJ-1, OJ-2, and OJ-3 by using PureLink Plant RNA Reagent (Life Technologies) according to the manufacturer’s protocols. The NEBNext Poly(A) mRNA Magnetic Isolation Module (NEB, E7490) was used to enrich the mRNA by isolating intact poly(A) + RNA from the previously isolated total RNA. Enriched mRNA was used as a template, and a sample library was constructed with the NEBNext mRNA Library Prep Master Mix Set for Illumina (NEB, E6110) and NEBNext Multiplex Oligos for Illumina (NEB, E7500). The constructed library was subjected to 1.8% agarose gel electrophoresis to determine the library insertion fragment size^71^. Then, a Library Quantification Kit-Illumina GA Universal (Kapa, KK4824) was used for quantification. A qualified library was used to form clusters in an Illumina cbot. Finally, sequencing was performed by Biomarker Biotechnology Corporation (Beijing, China) using an Illumina HiSeq 2500 (San Diego, CA, USA).

De novo transcriptome assembly was performed by Trinity^23^. The raw data generated by double-end sequencing were evaluated, filtered, and assembled, and then the unigene library was obtained. Based on the unigene library, gene structure annotation, gene expression analysis and gene functional annotation were performed. The prediction of the open-reading frames (ORFs) was performed by Getorf software (http://emboss.sourceforge.net/apps/cvs/emboss/apps/getorf.html).

### Differential expression analysis

The reads generated by transcriptome sequencing were aligned to the unigene library by Bowtie^72^. Based on the alignment results, RSEM was used to quantify transcript abundances. RSEM is an accurate and user-friendly software tool for quantifying transcript abundances from RNA-Seq data, and it does not rely on the existence of a reference genome^73^. To eliminate the impact of molar concentration and transcript length, RPKM (reads per kilobase of exon model per million mapped reads) was adopted to quantify gene expression^74^. Identification of DEGs was performed by EBSeq with FDR < 0.01 and fold change ≥2^75^.

### Library preparation, sequencing, and analysis for small RNA sequencing

The Illumina TruSeq Small RNA Library Prep Kit was used for library preparation. With total RNA as the input, the microRNA was then ligated with 5’ and 3’ adapters and used for reverse transcription. After PCR amplification was conducted, the target fragments from PAGE gel electrophoresis were recovered to create a cDNA library. Sequencing was performed on an Illumina HiSeq 2500 after sample testing.

Adapter sequences, low-quality reads, and sequences that were too long or too short were filtered from the raw data. The remaining high-quality reads were named clean reads and mapped to GenBank, the Rfam database^76^ and the transcriptome for sRNA annotation. miRDeep2^77^ software was used to analyze the clean reads, to identify miRNAs and to determine their expression. An authentic secondary structure of the precursor is considered one of the vital factors of a miRNA candidate, so we employed the Mfold^78^ program to predict the secondary structures of the miRNA precursors. Then, target prediction and DEG analysis were performed. For target prediction, identified miRNAs in *O. javanica* were used as query sequences against *O. javanica* transcriptome data by Targetfinder (http://carringtonlab.org/resources/targetfinder)^79^. Hits with <4 scores were chosen as candidate targets.

### Library preparation, sequencing, and analysis for degradome sequencing

Library preparation was employed according to the protocol proposed by German et al.^80^. Sequencing was performed on an Illumina HiSeq 2500. The cleaved small RNA targets from degradome data were predicted using CleaveLand^29^.

### Functional annotation

For functional annotations, all unigenes were mapped to the NCBI non-redundant nucleotide database (Nt), NCBI non-redundant protein database (Nr), computer-annotated supplement to Swiss-Prot (TrEMBL), annotated protein sequence database (Swiss-Prot) (http://www.expasy.ch/sprot), and Clusters of Orthologous Groups (COG) of proteins database (http://www.ncbi.nlm.nih.gov/COG)^81^ by BLASTx. Gene Ontology (GO) (http://www.geneontology.org) was adopted to map the functional assignments. GO annotation based on molecular function, biological process, and cellular component ontology was obtained by Blast2GO^82^. After GO classification, a GO tree was drawn using WEGO (http://wego.genomics.org.cn/cgibin/wego/index.pl). Based on the KEGG database (http://www.genome.jp/kegg), pathway assignments were performed by the online KEGG automatic annotation server (KAAS)^83^. Finally, the best matches were used to identify coding regions and to determine the sequence direction.

## Data Availability

All the raw data, as well as genome sequences, protein sequences, CDSs, and gene annotations, can be found in the Water dropwort database (http://apiaceae.njau.edu.cn/waterdropwortdb). All other data are available from the corresponding author upon reasonable request.

## Supplementary Figure

Supplementary Figure
Supplementary Tables

## References

[1] Naruhashi N, Iwatsubo Y (1998). Chromosome numbers and distributions of *Oenanthe javanica* (Umbelliferae) in Japan. J. Phytogeorg. Taxon..

[2] Sun BY, Park JH, Kwak MJ, Kim CH, Kim KS (1996). Chromosome counts from the flora of Korea with emphasis on Apiaceae. J. Plant Biol..

[3] Zhao D, Yan ZM, Zhang SN, Li JX, Liu HJ (2010). Karyotype analysis of main Umbelliferous vegetables. Acta Bot. Boreal-Occident. Sin..

[4] Li MY (2020). The genome sequence of celery (*Apium graveolens* L.), an important leaf vegetable crop rich in apigenin in the Apiaceae family. Hortic. Res..

[5] Que F (2019). Advances in research on the carrot, an important root vegetable in the Apiaceae family. Hortic. Res..

[6] Li MY (2018). Advances in the research of celery, an important Apiaceae vegetable crop. Crit. Rev. Biotechnol..

[7] Kim JY (2009). *Oenanthe javanica* extract accelerates ethanol metabolism in ethanol-treated animals. BMB Rep..

[8] Han YQ, Huang ZM, Yang XB, Liu HZ, Wu GX (2008). In vivo and in vitro anti-hepatitis B virus activity of total phenolics from *Oenanthe javanica*. J. Ethnopharmacol..

[9] Ma CJ (2010). Persicarin from water dropwort (*Oenanthe javanica*) protects primary cultured rat cortical cells from glutamate-induced neurotoxicity. Phytother. Res..

[10] Ma G (2007). The flavonoid component isorhamnetin in vitro inhibits proliferation and induces apoptosis in Eca-109 cells. Chem. Biol. Interact..

[11] Ku SK, Han MS, Bae JS (2013). Down-regulation of endothelial protein C receptor shedding by persicarin and isorhamnetin-3-O-galactoside. Thromb. Res..

[12] Jiang Q (2015). *De novo* transcriptome assembly, gene annotation, marker development, and miRNA potential target genes validation under abiotic stresses in *Oenanthe javanica*. Mol. Genet. Genomics.

[13] Jiang Q (2014). Effects of abiotic stresses on the expression of *Lhcb1* gene and photosynthesis of *Oenanthe javanica* and *Apium graveolens*. Biol. Plant..

[14] Jiang Q (2014). Selection of suitable reference genes for qPCR normalization under abiotic stresses in *Oenanthe javanica* (BI.) DC. PLoS ONE.

[15] Najla S, Sanoubar R, Murshed R (2012). Morphological and biochemical changes in two parsley varieties upon water stress. Physiol. Mol. Biol. Plants.

[16] Striker GG, Casas C, Manzur ME, Ploschuk RA, Casal JJ (2014). Phenomic networks reveal largely independent root and shoot adjustment in waterlogged plants of *Lotus japonicus*. Plant Cell Environ..

[17] Qi B, Yang Y, Yin Y, Xu M, Li H (2014). *De novo* sequencing, assembly, and analysis of the Taxodium ‘Zhongshansa’ roots and shoots transcriptome in response to short-term waterlogging. BMC Plant Biol..

[18] Zhu JK (2002). Salt and drought stress signal transduction in plants. Annu. Rev. Plant Biol..

[19] Sun X (2014). Advances in identification and validation of plant microRNAs and their target genes. Physiol. Plant..

[20] Wang C (2013). Characterization of target mRNAs for grapevine microRNAs with an integrated strategy of modified RLM-RACE, newly developed PPM-RACE and qPCRs. J. Plant Physiol..

[21] Shamimuzzaman M, Vodkin L (2012). Identification of soybean seed developmental stage-specific and tissue-specific miRNA targets by degradome sequencing. BMC Genomics.

[22] Luo RB (2012). SOAPdenovo2: an empirically improved memory-efficient short-read de novo assembler. Gigascience.

[23] Grabherr MG (2011). Trinity: reconstructing a full-length transcriptome without a genome from RNA-Seq data. Nat. Biotechnol..

[24] Kötting O (2005). Identification of a novel enzyme required for starch metabolism in Arabidopsis leaves. The phosphoglucan, water pikinase. Plant Physiol..

[25] Nylander M, Svensson J, Palva ET, Welin BV (2001). Stress-induced accumulation and tissue-specific localization of dehydrins in *Arabidopsis thaliana*. Plant Mol. Biol..

[26] Lee SC (2005). Characterization of an abiotic stress-inducible dehydrin gene, *OsDhn1*, in rice (*Oryza sativa* L.). Mol. Cells.

[27] Chan ZL (2012). Expression profiling of ABA pathway transcripts indicates crosstalk between abiotic and biotic stress responses in Arabidopsis. Genomics.

[28] Grondin A, Mauleon R, Vadez V, Henry A (2016). Root aquaporins contribute to whole plant water fluxes under drought stress in rice (*Oryza sativa* L.). Plant Cell Environ..

[29] Addo-Quaye C, Miller W, Axtell MJ (2009). CleaveLand: a pipeline for using degradome data to find cleaved small RNA targets. Bioinformatics.

[30] Ma Z (2014). Arabidopsis miR171-targeted scarecrow-like proteins bind to GT cis-elements and mediate gibberellin-regulated chlorophyll biosynthesis under light conditions. PLoS Genet..

[31] Liu ZW, Li H, Liu JX, Wang Y, Zhuang J (2020). Integrative transcriptome, proteome, and microRNA analysis reveals the effects of nitrogen sufficiency and deficiency conditions on theanine metabolism in the tea plant (*Camellia sinensis*). Hortic. Res..

[32] Kaessmann H (2010). Origins, evolution, and phenotypic impact of new genes. Genome Res..

[33] Iorizzo M (2016). A high-quality carrot genome assembly provides new insights into carotenoid accumulation and asterid genome evolution. Nat. Genet..

[34] Song XM (2020). Deciphering the high-quality genome sequence of coriander that causes controversial feelings. Plant Biotechnol. J..

[35] Song XM (2020). The celery genome sequence reveals sequential paleo-polyploidizations, karyotype evolution, and resistance gene reduction in Apiales. Plant Biotechnol. J..

[36] Han MV, Thomas GW, Lugo-Martinez J, Hahn MW (2013). Estimating gene gain and loss rates in the presence of error in genome assembly and annotation using CAFE 3. Mol. Biol. Evol..

[37] Morales-Cruz A (2015). Distinctive expansion of gene families associated with plant cell wall degradation, secondary metabolism, and nutrient uptake in the genomes of grapevine trunk pathogens. BMC Genomics.

[38] Zhao MM (2021). The application of single-cell RNA sequencing in studies of autoimmune diseases: a comprehensive review. Clin. Rev. Allergy Immunol..

[39] Li JR (2021). The chromosome-based lavender genome provides new insights into Lamiaceae evolution and terpenoid biosynthesis. Hortic. Res..

[40] Li CS, Lin F, An D, Wang WQ, Huang RD (2017). Genome sequencing and assembly by long reads in plants. Genes.

[41] Ca LI HP, Mücke I (1997). History, overview and applications of mediated lignolytic systems, especially laccase-mediator-systems (Lignozym-process). J. Biotechnol..

[42] Miniussi M, Del Terra L, Savi T, Pallavicini A, Nardini A (2015). Aquaporins in *Coffea arabica* L.: identification, expression, and impacts on plant water relations and hydraulics. Plant Physiol. Biochem..

[43] de la Cruz J, Karbstein K, Woolford JL (2015). Functions of ribosomal proteins in assembly of eukaryotic ribosomes in vivo. Annu. Rev. Biochem.

[44] Chou YT, Lo KY (2019). Thallium(I) treatment induces nucleolar stress to stop protein synthesis and cell growth. Sci. Rep..

[45] Axtell MJ, Meyers BC (2018). Revisiting criteria for plant MicroRNA annotation in the era of big data. Plant Cell.

[46] Jovanović Ž, Stanisavljević N, Mikić A, Radović S, Maksimović V (2014). Water deficit down-regulates miR398 and miR408 in pea (*Pisum sativum* L.). Plant Physiol. Biochem..

[47] Hajyzadeh M, Turktas M, Khawar KM, Unver T (2015). miR408 overexpression causes increased drought tolerance in chickpea. Gene.

[48] Curaba J, Talbot M, Li ZY, Helliwell C (2013). Over-expression of microRNA171 affects phase transitions and floral meristem determinancy in barley. BMC Plant Biol..

[49] Rogers SO, Bendich AJ (1985). Extraction of DNA from milligram amounts of fresh, herbarium and mummified plant tissues. Plant Mol. Biol..

[50] Cantarel BL (2008). MAKER: An easy-to-use annotation pipeline designed for emerging model organism genomes. Genome Res..

[51] Korf I (2004). Gene finding in novel genomes. BMC Bioinforma..

[52] Stanke M (2006). AUGUSTUS: ab initio prediction of alternative transcripts. Nucleic Acids Res..

[53] Slater GS, Birney E (2005). Automated generation of heuristics for biological sequence comparison. BMC Bioinforma..

[54] Yandell M, Ence D (2012). A beginner’s guide to eukaryotic genome annotation. Nat. Rev. Genet..

[55] Eilbeck K, Moore B, Holt C, Yandell M (2009). Quantitative measures for the management and comparison of annotated genomes. BMC Bioinforma..

[56] Jones P (2014). InterProScan 5: genome-scale protein function classification. Bioinformatics.

[57] Hunter S (2012). InterPro in 2011: new developments in the family and domain prediction database. Nucleic Acids Res..

[58] Finn RD (2010). The Pfam protein families database. Nucleic Acids Res..

[59] Attwood TK (2012). The PRINTS database: a fine-grained protein sequence annotation and analysis resource-its status in 2012. Database.

[60] Letunic I, Doerks T, Bork P (2012). SMART 7: recent updates to the protein domain annotation resource. Nucleic Acids Res..

[61] Ashburner M (2000). Gene ontology: tool for the unification of biology. The Gene Ontology Consortium. Nat. Genet..

[62] Li L, Stoeckert CJ, Roos DS (2003). OrthoMCL: identification of ortholog groups for eukaryotic genomes. Genome Res..

[63] Riaño-Pachón DM, Ruzicic S, Dreyer I, Mueller-Roeber B (2007). PlnTFDB: an integrative plant transcription factor database. BMC Bioinforma..

[64] Hedges SB, Marin J, Suleski M, Paymer M, Kumar S (2015). Tree of life reveals clock-like speciation and diversification. Mol. Biol. Evol..

[65] Fu LM, Niu BF, Zhu ZW, Wu ST, Li WZ (2012). CD-HIT: accelerated for clustering the next-generation sequencing data. Bioinformatics.

[66] Edgar RC (2004). MUSCLE: a multiple sequence alignment method with reduced time and space complexity. BMC Bioinforma..

[67] Price MN, Dehal PS, Arkin AP (2009). FastTree: computing large minimum evolution trees with profiles instead of a distance matrix. Mol. Biol. Evol..

[68] Stein LD (2002). The generic genome browser: a building block for a model organism system database. Genome Res..

[69] Wang GL, Que F, Xu ZS, Wang F, Xiong AS (2016). Exogenous gibberellin enhances secondary xylem development and lignification in carrot taproot. Protoplasma.

[70] Wang GL (2015). Morphological characteristics, anatomical structure, and gene expression: novel insights into gibberellin biosynthesis and perception during carrot growth and development. Hortic. Res..

[71] Li MY, Wang F, Jiang Q, Ma J, Xiong AS (2014). Identification of SSRs and differentially expressed genes in two cultivars of celery (*Apium graveolens* L.) by deep transcriptome sequencing. Hortic. Res..

[72] Langmead B, Trapnell C, Pop M, Salzberg SL (2009). Ultrafast and memory-efficient alignment of short DNA sequences to the human genome. Genome Biol..

[73] Li B, Dewey CN (2011). RSEM: accurate transcript quantification from RNA-Seq data with or without a reference genome. BMC Bioinforma..

[74] Mortazavi A, Williams BA, McCue K, Schaeffer L, Wold B (2008). Mapping and quantifying mammalian transcriptomes by RNA-Seq. Nat. Methods.

[75] Leng N (2013). EBSeq: an empirical Bayes hierarchical model for inference in RNA-seq experiments. Bioinformatics.

[76] Burge SW (2013). Rfam 11.0: 10 years of RNA families. Nucleic Acids Res..

[77] Friedländer MR, Mackowiak SD, Li N, Chen W, Rajewsky N (2012). miRDeep2 accurately identifies known and hundreds of novel microRNA genes in seven animal clades. Nucleic Acids Res..

[78] Zuker M (2003). Mfold web server for nucleic acid folding and hybridization prediction. Nucleic Acids Res..

[79] Fahlgren N (2007). High-throughput sequencing of Arabidopsis microRNAs: evidence for frequent birth and death of MIRNA Genes. PLoS ONE.

[80] German MA, Luo S, Schroth G, Meyers BC, Green PJ (2009). Construction of parallel analysis of RNA ends (PARE) libraries for the study of cleaved miRNA targets and the RNA degradome. Nat. Protoc..

[81] Tatusov RL (2003). The COG database: an updated version includes eukaryotes. BMC Bioinforma..

[82] Conesa A (2005). Blast2GO: a universal tool for annotation, visualization and analysis in functional genomics research. Bioinformatics.

[83] Moriya Y, Itoh M, Okuda S, Yoshizawa AC, Kanehisa M (2007). KAAS: an automatic genome annotation and pathway reconstruction server. Nucleic Acids Res..

