# Supplementary material for: Integrative genome, transcriptome, microRNA, and degradome analysis of water dropwort (Oenanthe javanica) in response to water stress
Source: Hortic Res. 2021 Dec 1;8:262. doi: 10.1038/s41438-021-00707-8 (PMC8633011; doi:10.1038/s41438-021-00707-8)
Supplement: Supplementary file 1 — Supplementary Figure [file 41438_2021_707_MOESM1_ESM.docx]

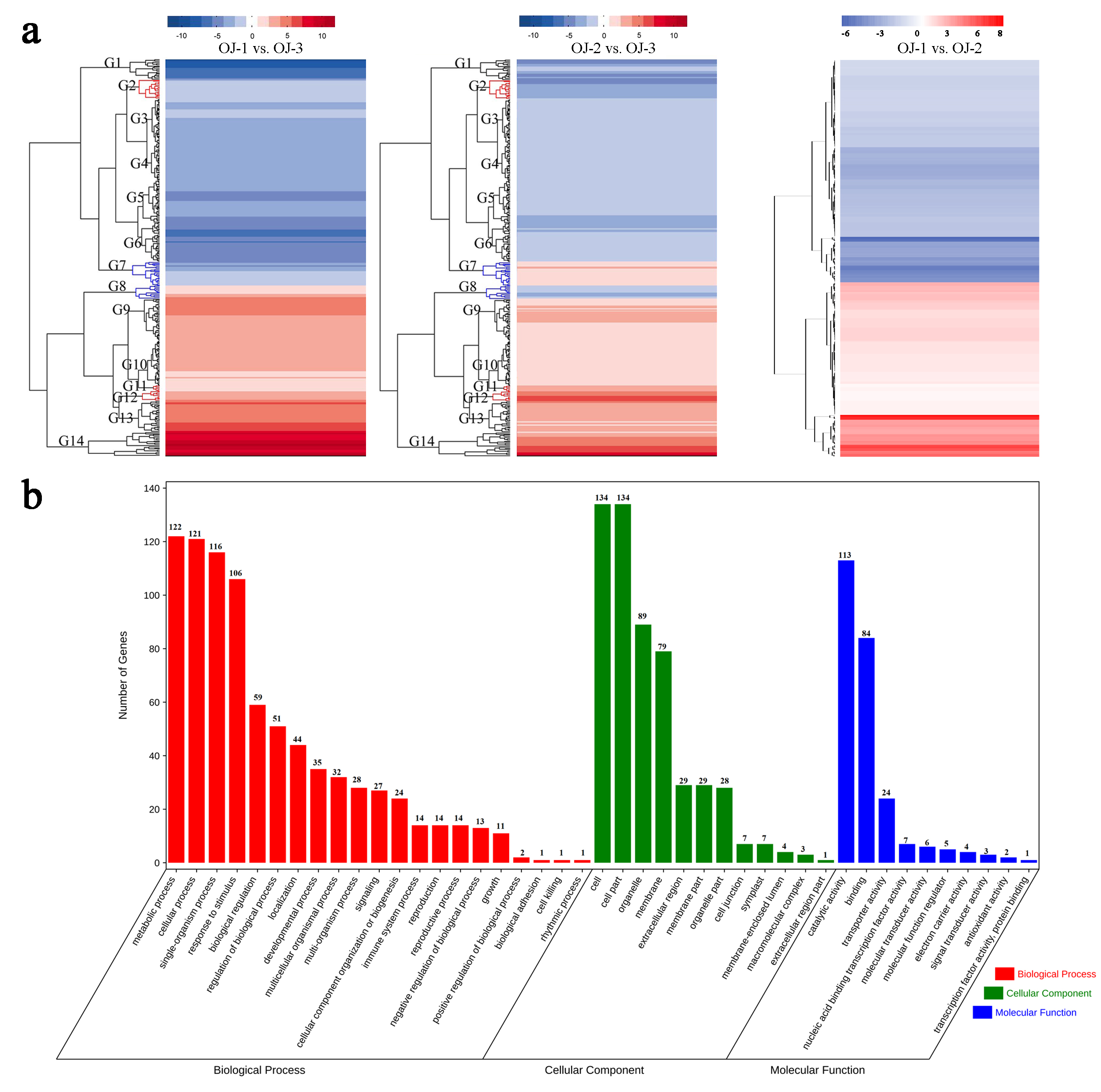


**Supplementary Fig. 1** The analysis of 333 differentially expressed genes in *O. javanica* among three water conditions.

(**a**) Hierarchical clustering of 333 DEGs. G means group; Red branch is G2, G11 and G12; Blue branch is G7 and G8; OJ-1 vs. OJ-3: log of RPKM_(OJ−1)_/RPKM_(OJ−3)_; OJ-2 vs. OJ-3: log of RPKM_(OJ−2)_/RPKM_(OJ−3)_; OJ-1 vs. OJ-2: log of RPKM_(OJ−1)_/RPKM_(OJ−2)_. (**b**) GO enrichment analysis of 333 DEGs in *O. javanica* among three different water conditions.
